# Hospital Meetings

**Published:** 1896-05-23

**Authors:** 


### HOSPITAL MEETINGS.

### NORTH-EASTERN HOSPITAL FOR CHILDREN.

The North-Eastern Hospital for Children is doing a good work in the poverty-stricken and thickly-populated portion of the East-end of London in which it is situated. The committee are ambitious, however, and wish to do more. It was this, probably, which led the treasurer (Mr. J. Lister Godlee), who presided at the annual meeting on the 13th inst., to point out that the hospital had sufficient land adjoining the present premises to allow of its enlargement. Unfortunately, too, they had, he said, the patients to use any addition which might be made to the accommodation. Enlargement, however, meant a considerable sum of money, and although they had the nucleus of a fund in the £5,000 remaining out of the legacy received from the late Mr. Horniman, they still required about £10,000 before they could commence work. Dealing with the financial position of the hospital, Mr. Godlee said that during the past year it had decidedly improved. Chiefly through the legacies received, the mortgage (which has gradually accumulated during adverse years) was reduced by £1,500, leaving it now £3,000. To pay off this debt the committee have organised a bazaar to be held in June at the Queen's Hall. But they appeal for still further funds, namely, for £2 500, to enable the hospital to pay its way this year, and for £500 more in annual subscriptions in order to give the hospital a fair chance of keeping out of debt in the future.

### EAST LONDON HOSPITAL FOR CHILDREN.

One of the first of the charity dinners held at the recently-opened Hotel Cecil was that of the East London Hospital for Children (on the 13th inst), a hospital which works in one of the poorest districts of the metropolis. Mr. John Aird, M.P., who presided, after referring in appreciative terms to the staff of the hospital, said that the hospital had been greatly benefited by the extensions made last year, but over £1,000 was needed to pay for all the improvements made. Funds were also needed for a further development of the charity in the shape of a convalescent home at Bognor, towards which they had already received about £3,500. Mr. H. W. Trinder said that the hospital required a further £1,000 for the erection of the convalescent home, and £500 to £600 a year for its maintenance. The East London Hospital was recognised as a very good school for nursing, and it was proposed to still futher develop this branch of its work. Cardinal Vaughan described the hospital as one of the greatest charities in the East-end; he knew the poor were well cared for when they sought its aid, and the services rendered by the nurses left nothing to be desired.—Donations amounting to about £1,250 were received as the result of the dinner.—The annual meeting was held at the hospital on the 14th inst.

### METROPOLITAN HOSPITAL.

The annual meeting of the Metropolitan Hospital was held on the 18th inst., Mr. C. J. Thomas in the chair. The hospital is situated in the Kingsland Road, N.E., close to two of the poorest districts in London—Hoxton and Shoreditch—the dwellers in which sorely require the help which the hospital has to give, a fact which is amply testified to by the numbers of patients treated. In 1895, 971 in and 21,340 out-patients were under treatment, a great increase on those attending in 1894, viz., 781 and 16,033 respectively. The total expenditure was £10,906, of which £148 was expended on extraordinary buildings and repairs, and £637 on the Provident Department. To meet this expenditure the hospital only received £9,402, of which £772 came from legacies. The convalescent home given by Mr. J. Passmore Edwards will shortly be erected on a site given by Mr. F. S. W. Cornwallis, near Staplehurst, in Kent. A successful concert in aid of the hospital was held at the St. James's Hall on the 15th inst., the programme being rendered throughout in a most creditable manner. Several of the part songs by the Magpie Madrigal Society were of the 16th and 17th centuries, a particularly charming one being "Aime Moi, Bergère," by Jacques Lefevre (1613). Modern songs were very pleasingly performed by Mr. Theodore Byard and Miss Louise Phillips, while Mr. Louis D'Egville's violin solos were played with great feeling. The concert realised between £60 and £70.

### CHARING CROSS HOSPITAL.

An interesting ceremony took place on the 13th inst. in the Victoria Ward, Charing Cross Hospital, in connection with the formal opening of a cot presented by Miss Maitland-Erskine (a former sister of the hospital), and endowed by the Ministering Children's League. There was a large gathering of ladies and gentlemen, including Lady Harbord, Mr. George Drummond (the treasurer), Mr. Percy Borrett (the chairman) and Mrs. Borrett, Dr. and Mrs. Bruce, and several members of the staff. After dedicatory prayers had been said by the Chaplain, the Treasurer addressed the company, and thanked the members of the league for their support in a time of great need. Subsequently the first little patient was placed in the cot, which is a very handsome one, painted in white and gold, with sliding sides.

### LONDON SOCIETY FOR TEACHING THE BLIND TO READ.

The Lord Chief Justice of England (Lord Russell of Killowen) presided at the festival dinner of the above society, which was held at the King's Hall, Holborn Restaurant, on the 18th inst. This is the first social festival which has been held in connection with the society for over a quarter of a century, the committee feeling that now owing to the failure of annual subscriptions, and the inroads made upon the capital, a special effort was necessary. The objects of the society are first to teach the blind the ordinary rudiments of education, and secondly to impart to them instruction in useful industries, by means of which when they have left the care of the institution they may be able to support themselves. The contributions received amounted to £1,338.

